# Fertility counseling experiences and perceived gaps in provider support among people living with HIV: a qualitative study from an antiretroviral therapy clinic in Ghana

**DOI:** 10.3389/frph.2026.1914832

**Published:** 2026-08-18

**Authors:** Victor Luckyboy Dzramado, Priscilla Asantewaa Boadi

**Affiliations:** 1 Department of Biostatistics, Cape Coast Teaching Hospital, Cape Coast, Ghana; 2 St. Michael Nursing and Midwifery Training College, Kumasi, Ghana

**Keywords:** fertility counseling, Ghana, people living with HIV, qualitative research, reproductive health, safer conception, thematic analysis

## Abstract

**Introduction:**

Antiretroviral therapy (ART) has made safer childbearing achievable for people living with HIV (PLHIV), and the reproductive health counseling PLHIV receive within HIV care shapes whether they can act safely on their fertility intentions. This study explored, from the perspective of PLHIV themselves, how the reproductive counseling they reported receiving shaped their fertility decision making, and which aspects of counseling support they perceived to be missing.

**Methods:**

A qualitative descriptive design with inductive reflexive thematic analysis was used. Twelve PLHIV (nine women, three men) distributed across the 26–50 year age bands and receiving ART at St. Michael's Hospital ART clinic, Pramso, Ashanti Region, were recruited through maximum variation purposive sampling within criterion based eligibility. In depth semi structured interviews were conducted between August and October 2024, audio recorded, transcribed verbatim, and coded by both authors, with differences resolved by consensus. Reporting followed the Consolidated Criteria for Reporting Qualitative Research.

**Results:**

Three themes were developed. First, participants reported clear and reassuring education on prevention of vertical (mother to child) transmission, while describing little guidance on conceiving safely with a partner of a different HIV status. Second, participants described providers as warm and accessible, yet experienced the counseling itself as general rather than matched to their own circumstances. Third, participants reported reliable treatment access and routine reproductive education, but described few services beyond education and basic counseling.

**Discussion:**

Participants perceived gaps in counseling support, particularly beyond prevention of vertical transmission. These are reported experiences and do not measure counseling quality or provider knowledge, and they may equally reflect consultation time, service organization, referral availability, and the faith based institutional context. The findings indicate areas that participants themselves identified for strengthening, including safer conception counseling, individualized fertility planning, and clearer referral pathways.

## 1 Introduction

People living with HIV (PLHIV) now live longer, healthier lives because of widespread access to antiretroviral therapy (ART), and an increasing proportion express the desire to have children (1, 2). Sustained viral suppression on ART preserves health and eliminates the risk of sexual transmission of HIV to a partner, a principle widely summarized as Undetectable equals Untransmittable, and it enables conception and pregnancy with very low risk of transmission to the infant when appropriate interventions are used (2, 3). In Ghana, the scale up of ART has been accompanied by improving treatment coverage and declining HIV related mortality, shifting the experience of HIV toward that of a long term condition compatible with family formation (4, 5). As survival and quality of life have improved, fertility desires and intentions among PLHIV have risen across sub Saharan Africa, including among women and men receiving ART (6, 7).

This shift places reproductive health counseling at the center of comprehensive HIV care. Two concepts recur in this study and are defined here at first use. Safer conception refers to the set of methods that allow a person or couple living with HIV to attempt pregnancy while minimizing the risk of HIV transmission to a partner and to the infant, including timed condomless intercourse during viral suppression, pre exposure prophylaxis for an HIV negative partner, self-insemination, and, where available, sperm washing. A serodifferent couple, a term now preferred over the older word serodiscordant, is a partnership in which one partner is living with HIV and the other is HIV negative. The term serodifferent is used consistently throughout this article.

The way people form fertility intentions can be understood through the Theory of Planned Behavior, which holds that intentions are shaped by attitudes toward the behavior, subjective norms, and perceived behavioral control, that is, a person's belief that they can successfully and safely carry out the behavior (8). In this study the theory served as a sensitizing lens for framing the topic and for interpreting the findings in the discussion. It was not applied as a coding framework, and the analysis itself was inductive, as described in Section 2.6. Viewed through this lens, attitudes toward childbearing with HIV are shaped by beliefs about transmission and health, subjective norms reflect strong pronatalist expectations, and perceived behavioral control depends partly on whether counseling provides accurate, actionable information about how to conceive and give birth safely. Reproductive counseling is therefore closely bound up with fertility decision making (9, 10).

Early work in the region established that HIV positive women and men actively consider childbearing and that their reproductive needs are frequently overlooked within HIV services (9–11). Evidence from across sub Saharan Africa also indicates that reproductive counseling for PLHIV is often incomplete, particularly beyond prevention of vertical transmission. A study at two HIV clinics in Malawi documented that providers held generally supportive attitudes toward childbearing but lacked accurate knowledge of safer conception methods (12). Among 57 Ugandan HIV providers, knowledge, self-efficacy, and intentions to provide safer conception counseling were limited, even though childbearing desires were reported by 59 percent of clients and approximately 50 percent of clients were in serodifferent partnerships (13). A nationally representative survey of 5,198 women living with HIV attending 245 HIV clinics in Uganda found that although overall awareness of any safer conception method reached 74.1 percent, only 13.2 percent could identify three or four methods and 42 percent knew only a single method (14). In a separate sample of 400 Ugandan clients with fertility intentions, just over half were aware that manual self-insemination and timed unprotected intercourse reduce transmission risk during conception, and only about 15 percent were aware of sperm washing and pre exposure prophylaxis (15). Where structured safer conception services have been implemented, PLHIV adopt these strategies, with one South African demonstration clinic reporting continuation of self-insemination at 69.1 percent and timed condomless sex at 75.4 percent of follow up visits (16). A systematic review of family planning counseling for women living with HIV in low and middle income countries concluded that such counseling improves contraceptive uptake and reproductive intentions but remains inconsistently delivered (17).

In Ghana, reproductive health services for PLHIV remain incompletely integrated, and unmet needs for modern contraception and broader reproductive counseling persist (18). Strong pronatalist and patriarchal norms intensify the pressure to bear children, particularly for women, which raises the stakes of receiving accurate and complete information (8, 19). Studies from the Ashanti Region, where the present study was conducted, have documented substantial unmet need for family planning and gaps in reproductive counseling among women living with HIV at facilities in Kumasi, and have shown that these women prefer to discuss reproductive concerns with their HIV clinicians (20–22). This regional evidence base is largely quantitative and focused on contraception, and few Ghanaian studies have used patient narratives to examine how PLHIV themselves experience the counseling they receive and where they perceive it to fall short.

The present study addressed this gap and pursued two distinct but related aims. The first aim was to explore how the reproductive health counseling that PLHIV reported receiving shaped their fertility intentions and decision making. The second aim was to identify which aspects of reproductive counseling and support participants perceived to be missing or insufficient within HIV care at their clinic. Data were collected only from patients. The study therefore reports patients’ experiences and perceptions, and it does not measure counseling quality, provider knowledge, or provider training.

## 2 Materials and methods

### 2.1 Study design

The study used a qualitative descriptive design with inductive reflexive thematic analysis (23–25). Qualitative description is well suited to producing a rich, data near account of participants’ experiences and perceptions of a defined phenomenon, in this case reproductive counseling within HIV care, and it stays close to participants’ own accounts rather than moving toward heavy theoretical abstraction (23). Reflexive thematic analysis was selected because the study sought patterned meanings across the whole dataset rather than a detailed reconstruction of individual lifeworlds (24, 25). The design, research questions, analysis, and reflexivity all follow the principles of qualitative description and thematic analysis. Reporting followed the Consolidated Criteria for Reporting Qualitative Research (26).

### 2.2 Study setting

The study was conducted at the ART clinic of St. Michael's Hospital, a district level Catholic mission referral facility located at Pramso in the Bosomtwe District of the Ashanti Region, Ghana, approximately 20 kilometers from Kumasi. The clinic operates within Ghana's national HIV treatment program and provides free ART, CD4 and viral load monitoring, opportunistic infection management, and counseling services. At the time of the study, approximately 120 PLHIV were registered in the ART program and received regular follow up care. The facility is owned and administered within the Catholic Health Service of Ghana. Its faith based character forms part of the institutional context in which counseling was delivered and is revisited in the discussion and limitations.

### 2.3 Eligibility criteria and rationale for participant selection

Inclusion criteria were: aged 18 years or older; a confirmed diagnosis of HIV; currently receiving ART at the study clinic; attending the clinic for at least one prior follow up visit, so that participants had some experience of routine clinic counseling on which to draw; able to communicate in English or Twi; and willing and able to give written informed consent.

Exclusion criteria were: aged under 18 years; acutely unwell or in physical distress on the day of the clinic visit; presenting with evident cognitive impairment or any condition that would impede informed consent or sustained interview participation; newly enrolled patients attending for a first visit only; and any patient who declined consent.

Participants were selected to obtain a sample capable of speaking to reproductive counseling from a range of life positions. Because fertility decision making differs by sex, relationship status, and parenting history, selection deliberately sought variation on these characteristics rather than a homogeneous group, so that the dataset would contain both those actively weighing future childbearing and those who regarded their families as complete.

### 2.4 Sampling and recruitment

Maximum variation purposive sampling was used, within the criterion based eligibility above, to obtain a sample varying in age, sex, marital status, educational background, and parenting experience.

Recruitment proceeded in a stepwise manner. During routine clinic visits, clinic nurses and counselors who formed part of participants’ usual care team screened attending patients against the eligibility criteria. The nurse briefly introduced the study and asked whether the patient was willing to hear more about it. Patients who agreed were then introduced to the first author, a nurse midwife educator who was not a member of their clinical care team and had no role in their treatment decisions, so that the decision to participate was separated from the ongoing care relationship. The first author explained the purpose, procedures, voluntary nature of participation, the absence of any effect on care, and the right to withdraw at any time, and then obtained written informed consent. Interviews were scheduled at the participant's convenience and were conducted either on the same clinic day, after the clinical consultation had ended, or on a subsequent visit. Participants received no monetary compensation but were reimbursed for transportation costs.

A small number of eligible patients approached during the recruitment period declined to participate, most commonly citing time constraints or reluctance to discuss fertility matters. Declines were noted informally rather than counted systematically, so an exact figure is not available, and this is acknowledged in the limitations. Repeat invitations were not issued to patients who declined.

Recruitment continued until data saturation was judged to have been reached. Saturation was assessed jointly by both authors through debriefing after each interview and review of the developing codebook. Code saturation, defined as the point at which no new codes emerged, was reached at approximately the ninth to tenth interview, and meaning saturation was confirmed by the eleventh and twelfth interviews, which reinforced rather than expanded the existing themes (27, 28). The final sample comprised 12 participants, nine women and three men, distributed across the 26–50 year age bands.

### 2.5 Data collection

Data were collected through in depth, semi structured individual interviews conducted between 4 August 2024 and 5 October 2024.

The interview guide was developed from the two research aims and the wider literature on fertility among PLHIV. A draft was reviewed by the research supervisor for clarity, relevance, and non-leading phrasing, and revised accordingly. The revised guide was piloted with two clinic attendees who met the eligibility criteria; these pilot interviews were used to check question comprehension, wording in Twi, and sequencing, and are not included in the analyzed dataset. Piloting led to simplification of wording on two items and the addition of probes inviting concrete examples. Probing continued to be refined during data collection as participants raised topics not anticipated in the guide, while the core questions remained unchanged.

The guide invited participants to describe their experience of HIV care and services, their thoughts and feelings about having children, the factors shaping their reproductive decisions, how providers addressed their fertility concerns, and any counseling they had received relating to fertility and HIV. Example items included, “How would you describe your access to and experience with health care services?”, “How do health care providers address your fertility concerns?”, and “Have you received any counseling or support specifically related to fertility and HIV?”, each followed by open probes inviting elaboration and examples. The complete interview guide is provided as Supplementary Material S1.

Interviews were conducted in English or Twi according to participant preference, in a private consulting room within the hospital, with only the participant and the interviewer present. Each interview lasted between 45 and 90 min and was conducted in a single session. Interviews were audio recorded with permission and transcribed verbatim; interviews conducted in Twi were transcribed and then translated into English by the first author, who is fluent in both languages, and the translations were checked against the recordings for accuracy of meaning. Field notes documenting contextual observations and non-verbal cues supplemented the transcripts. Transcripts were not returned to participants for comment.

The guide did not contain a rating scale. When invited to describe their experience of services, some participants spontaneously offered an approximate percentage to summarize their satisfaction, for example a figure of about 70 percent. These self-generated figures are reported as participants’ own qualitative expressions of satisfaction and not as scores from any instrument administered by the research team.

### 2.6 Data analysis

Analysis followed the six phases of reflexive thematic analysis: familiarization, initial coding, generating initial themes, reviewing themes, defining and naming themes, and producing the report (24, 25). Coding was inductive and iterative. Codes were generated from the data rather than applied from a predetermined framework, and the coding frame was revisited repeatedly as later transcripts were read, with earlier transcripts recoded whenever the frame changed. The Theory of Planned Behavior informed the framing of the study and the interpretation of findings in the discussion, but was deliberately not used to structure coding.

The first author transcribed the interviews and coded all twelve transcripts manually, line by line, staying close to participants’ own language and using *in vivo* codes wherever possible. No qualitative data analysis software was used, and coding was recorded in a manual codebook with linked extracts. The second author independently read all twelve transcripts and independently coded a purposively selected subset of four, chosen to span sex, marital status, and parenting history. The two authors then compared codes extract by extract.

Interpretive differences were managed through structured discussion. Where the authors had applied different codes to the same extract, both codes were retained provisionally and tested against the wider dataset before a decision was made, rather than one being discarded at the point of disagreement. Where differences concerned the boundary between what a participant had described and what was being inferred, the more conservative, data near reading was adopted. Any difference that remained unresolved after discussion was referred to the research supervisor for adjudication. Focused codes were then grouped into candidate themes, which were reviewed against the coded extracts and against the full dataset for internal coherence and distinctiveness. Theme names were revised iteratively and finalized through team discussion so that each expressed the substance of what participants reported. The point of saturation identified during data collection was checked against the fully coded dataset, and the final interviews produced no new themes (27, 28). Throughout, the authors kept analytic memos and maintained an audit trail linking codes, extracts, and themes.

### 2.7 Reflexivity and positionality

Both authors are Ghanaian and brought professional and personal perspectives that shaped the research. The first author is a nurse midwife educator and the second works in biostatistics.

The authors identified several assumptions that could have influenced interpretation. The first author's midwifery training carries a professional commitment to comprehensive, individualized reproductive counseling, which created a predisposition to read general or routine counseling as deficient rather than as adequate for many patients’ needs. The second author's quantitative background created a tendency to look for patterns across cases and to treat convergence as significance, which risked flattening individual variation. Both authors also share the pronatalist cultural expectations common in Ghana, which could have led them to treat a desire for children as the default and to under attend to participants who did not want more children. These assumptions were recorded in bracketing memos at the outset and revisited during coding; the third assumption in particular prompted deliberate re-examination of the accounts of participants who considered their families complete, so that these were analyzed as substantive positions rather than as absence of data.

Power relations were considered at each stage. Participants were patients in a clinic on which they depended for treatment, and the first author is a health professional and an educator, an asymmetry that could have discouraged criticism of services. This was managed by recruiting through a researcher outside the clinical care team, stating repeatedly and explicitly that participation and responses would not affect care in any way, conducting interviews in private after clinical consultations had concluded, using participant codes from the point of transcription, and framing questions neutrally so that neither satisfaction nor dissatisfaction was signaled as the expected answer. The authors nonetheless recognize that this asymmetry cannot be eliminated, and its implications are addressed in the limitations.

Familiarity with the setting cut both ways. The first author's knowledge of Ghanaian ART clinic routines aided rapport, made Twi interviewing natural, and allowed participants’ references to clinic practice to be understood without lengthy explanation. The same familiarity risked normalizing what was routine and thereby overlooking it as a finding. The second author, who had no prior involvement with the study clinic, functioned deliberately as an outsider reader of the transcripts, and several codes concerning taken for granted clinic routine were generated through this contrast.

Strategies used to reduce interpretive bias were therefore: reflexive journaling by the first author throughout data collection and analysis; bracketing memos recording assumptions before coding began; analytic memos documenting each interpretive decision; independent coding by a second author with a different disciplinary lens; peer debriefing sessions between the authors after each interview and at each analytic phase; revisiting and recoding earlier transcripts whenever the coding frame changed; the deliberate search for accounts that contradicted the emerging pattern; and adoption of the more conservative reading wherever description and inference were in tension.

### 2.8 Trustworthiness

Trustworthiness was addressed through credibility, dependability, confirmability, and transferability (29, 30). Credibility was supported by verbatim transcription, prolonged engagement across the three month data collection period, peer debriefing between the two authors, checking of translated extracts against the recordings, and grounding interpretation in participants’ own words. Dependability was supported by a documented audit trail, a shared codebook, and independent second coder review of a purposive subset. Confirmability was supported by bracketing and analytic memos and by anchoring each theme in direct quotations, so that readers can trace interpretations back to the data. Transferability was supported by thick description of the setting, participants, and institutional context, allowing readers to judge applicability to comparable ART clinics.

### 2.9 Ethics statement

Ethical approval was obtained from the Committee on Human Research, Publication and Ethics at Kwame Nkrumah University of Science and Technology, College of Health Sciences, School of Dentistry (reference number CHRPE/AP/569/24), valid from 8 June 2024 to 7 July 2025. An introductory letter was received from Kwame Nkrumah University of Science and Technology, College of Health Sciences (reference NURS/INTRO/Vol. 1, 7 March 2024). Additional ethical clearance was secured from the Catholic Health Service Trust, Ghana (reference number SMH/RA.007/24, received on 6 June 2024), and permission was obtained from the administration of St. Michael's Hospital.

All participants provided written informed consent after receiving detailed information about the study's purpose, procedures, risks, benefits, and their right to withdraw at any time without any effect on their healthcare. Information was provided in English or Twi according to preference, and participants were given the opportunity to ask questions before signing.

Confidentiality was protected at every stage. Participant codes (P1–P12) replaced names from the point of transcription, and no names, addresses, or other direct identifiers were recorded on transcripts or field notes. The linking list between codes and identities was held separately from the data. Audio files and transcripts were stored on password protected devices, and access was restricted to the research team. Interviews were conducted in a private room out of hearing of other patients and staff, and no member of participants’ clinical care team was present. Reported extracts use participant codes only, and identifying details mentioned in passing were removed.

Because the interviews addressed sensitive matters including HIV status, sexual relationships, and childbearing, participant support arrangements were made in advance. Participants were told they could decline any question, pause, or stop the interview at any point without giving a reason. The interviewer monitored for signs of distress and was prepared to suspend the interview if distress arose. A pathway was arranged in advance with the hospital whereby any participant who became distressed or who raised an unmet clinical or psychosocial concern during the interview could be referred, with their consent, to the clinic counselor for follow up support on the same day. Participants received no monetary compensation but were reimbursed for transportation costs incurred through participation.

## 3 Results

### 3.1 Characteristics of participants

The twelve participants varied in age, marital status, education, employment, and parenting experience, as presented in Table 1. The sample comprised nine women and three men distributed across the 26–50 year age bands. Participants were single, married, divorced, or widowed, with educational attainment ranging from no formal education to tertiary level, and had between none and six children. All children born to participants were reported by participants as HIV negative.

**Table 1:** Characteristics of participants.

Participant code	Age group (years)	Sex	Marital status	Education level	Employment status	Number of children	Children's HIV status	Time since HIV diagnosis (years)^ᵃ^
P1	26–30	Female	Single	Tertiary	Student	0	N/A	NR
P2	36–40	Female	Divorced	Secondary	Unemployed	1	Negative	NR
P3	26–30	Female	Married	Tertiary	Employed	1	Negative	NR
P4	31–35	Male	Married	Secondary	Employed	2	Negative	NR
P5	41–45	Female	Widow	None	Unemployed	2	Negative	NR
P6	36–40	Female	Married	Tertiary	Employed	3	Negative	NR
P7	46–50	Male	Married	Tertiary	Employed	6	Negative	NR
P8	26–30	Female	Single	Tertiary	Student	0	N/A	NR
P9	46–50	Female	Married	Secondary	Employed	3	Negative	NR
P10	31–35	Female	Single	Tertiary	Employed	3	Negative	NR
P11	26–30	Male	Single	Secondary	Employed	0	N/A	NR
P12	31–35	Female	Married	Tertiary	Employed	2	Negative	NR

Time since diagnosis was not systematically documented at data collection and is not reported to avoid retrospective reconstruction. N/A, not applicable (participant had no children).

ᵃ NR, not recorded.

### 3.2 Overview of themes and representation across the sample

**Figure 1:**
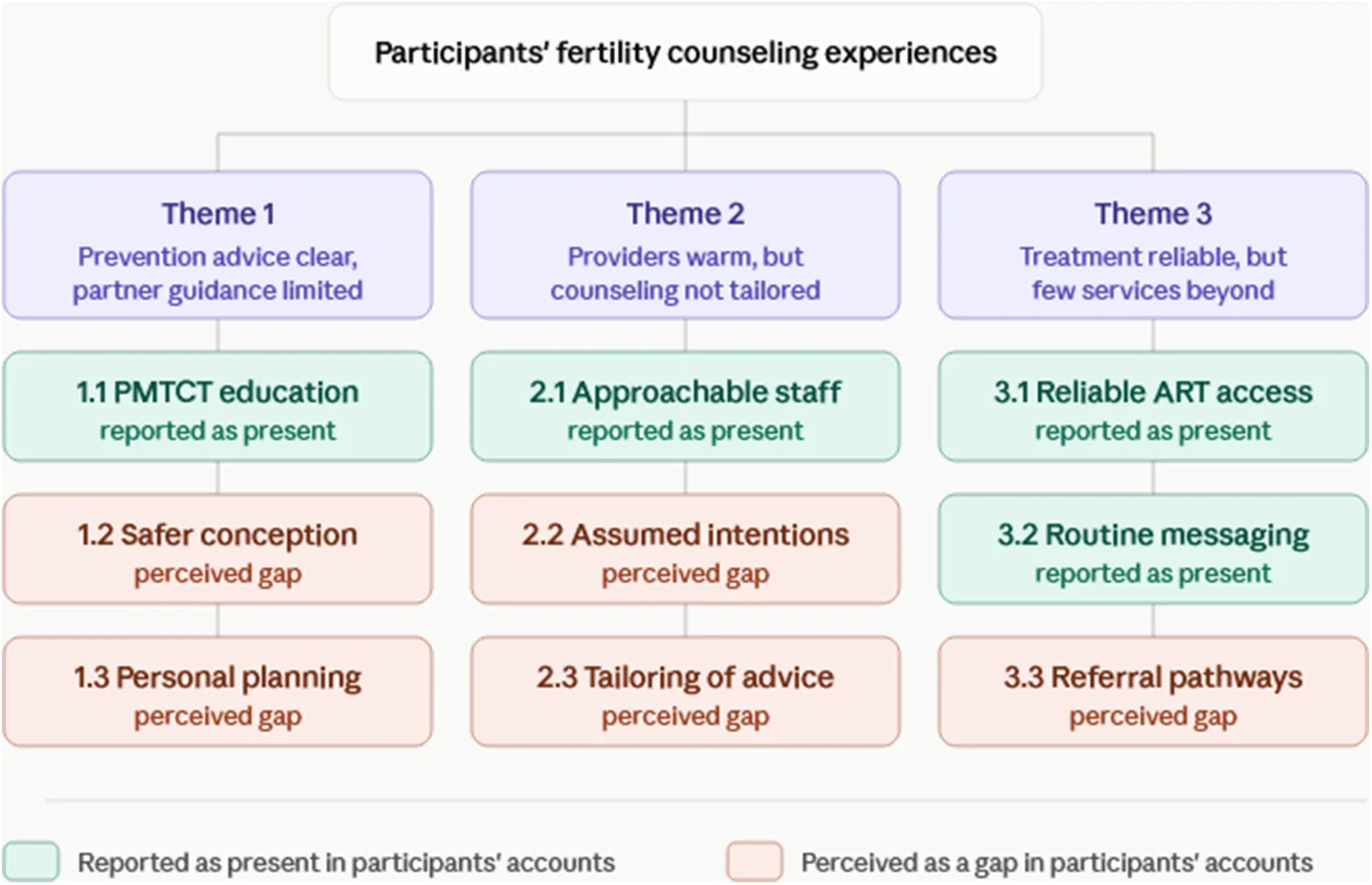
Thematic map of participants’ fertility counseling experiences, showing reported strength of prevention-of-transmission education alongside perceived gaps in safer conception, individualized, and specialized counseling.

Inductive reflexive thematic analysis produced three themes describing what participants reported about the reproductive counseling they received and what they perceived to be missing. The themes and sub-themes are summarized in Table 2, and their relationships are shown in Figure 1. Figure 1 was constructed from the coded dataset after theme development and represents the study's own findings; it is not a pre-existing conceptual model.

**Table 2:** Themes and sub-themes on participants’ fertility counseling experiences.

Theme	Sub-themes
Theme 1: Participants reported clear education on preventing transmission to the baby, but little guidance on conceiving safely with a partner	1.1 Clear and reassuring education on preventing vertical transmission; 1.2 Little reported guidance on safer conception for serodifferent couples; 1.3 Counseling experienced as general rather than matched to personal circumstances
Theme 2: Participants described providers as warm and accessible, while experiencing the counseling itself as not individualized	2.1 Providers described as approachable and encouraging; 2.2 Counseling experienced as pitched to assumed rather than actual intentions; 2.3 Fertility raised routinely, but without tailoring
Theme 3: Participants reported reliable treatment and routine education, but few services beyond basic counseling	3.1 Reliable ART access and reported health stability; 3.2 Reproductive messaging reported as part of routine visits; 3.3 Few reported services or referrals beyond education

Contribution to the themes was uneven across the sample, and this is reported rather than balanced artificially. Accounts relating to counseling content were most detailed among participants who were actively weighing future childbearing, particularly P2, P3, P6, P8, and P10. Participants who regarded their families as complete, notably P7 and P9, spoke to the themes chiefly by explaining their disengagement from counseling they felt did not apply to them, which is analyzed below as a substantive position rather than as missing data. P1, recently diagnosed, contributed a divergent account in which fertility counseling had not yet been offered.

Four participants, P4, P5, P11, and P12, contributed comparatively little to the counseling themes. Their interviews concentrated on diagnosis, treatment, disclosure, and livelihood concerns, and they said relatively little in response to the fertility counseling questions. Their sparse contribution is stated here explicitly rather than compensated for by drawing broader inferences from brief remarks. This uneven distribution means that the perspectives of two of the three male participants, and of the widowed participant, are underrepresented in the findings that follow, and this is carried forward into the limitations.

### 3.3 Theme 1: participants reported clear education on preventing transmission to the baby, but little guidance on conceiving safely with a partner

This theme describes what participants recalled being told about reproduction within HIV care. Illustrative accounts, with the interview question that prompted each and the situation the participant was describing, are presented in Table 3.

**Table 3:** Illustrative participant accounts on reported counseling content.

Sub-theme	Interview prompt	Participant's situation	Illustrative account	Participant
Clear and reassuring education on preventing vertical transmission	How do providers address your fertility concerns?	Single woman without children, anticipating future childbearing	“That having the disease doesn't deter one from not having kids and in our condition, we can still have healthy babies… every time I come for checkup.”	P8 (Female, Single)
Clear and reassuring education on preventing vertical transmission	What would motivate you to have a child in your condition?	Married woman with one child, considering another pregnancy	“I was told that you will be put on medications to protect the unborn baby from getting infected… It has changed my perspective on marriage and child bearing.”	P3 (Female, Married)
Little reported guidance on safer conception for serodifferent couples	How do providers address your fertility concerns?	Married woman describing counseling covering her partner as well as the baby	“There are drugs I can take to prevent my baby from having the disease and how I can get pregnant with my partner without infecting him as well.”	P6 (Female, Married)
Little reported guidance on safer conception for serodifferent couples	Have you received any counseling on fertility and HIV?	Single woman with three children, asked directly about fertility counseling	“Yes please, we are taught at the hospital after child bearing in our condition and our general wellbeing.”	P10 (Female, Single)
Counseling experienced as general rather than matched to personal circumstances	What are your reasons for wanting or not wanting more children?	Divorced woman who wants more children but has no current partner	“We are being taught every time I visit the hospital and I will rate them about 70%. I want to have more babies but I don't have a partner at the moment.”	P2 (Female, Divorced)
Counseling experienced as general rather than matched to personal circumstances	Are you taught at the hospital?	Married woman with three children who considers her family complete	“Yes we are taught but I don't need babies again so I don't really pay attention to it.”	P9 (Female, Married)
Divergent case: counseling not yet received	Have you received any counseling on fertility and HIV?	Recently diagnosed single woman, early in treatment	“Not yet, but I think along the line I will receive counseling on fertility issues.”	P1 (Female, Single)

Asked how providers addressed their fertility concerns, participants described education about protecting a future child from infection. P8, a single woman without children who was anticipating childbearing at some future point, said she was told at every check-up that having HIV did not prevent her from having children and that healthy babies were possible. P3, a married woman with one child who was considering another pregnancy, recalled being told that medication would be given to protect an unborn baby, and she linked this information directly to a change in how she thought about marriage and childbearing. These accounts of prevention focused education recurred across the interviews of participants who were considering childbearing.

Accounts extending beyond transmission to the baby were far fewer. P6, a married woman, was the only participant whose account described counseling that also covered protecting a partner, saying she had learned both about medication to prevent the baby acquiring the disease and about becoming pregnant without infecting her partner. By contrast, P10, a single woman with three children, was asked directly whether she had received counseling on fertility and HIV, and described teaching about childbearing and general wellbeing without any reference to a partner. Across the dataset, references to specific safer conception methods, whether timed intercourse, pre exposure prophylaxis for a partner, self-insemination, or sperm washing, did not appear in participants’ accounts, apart from P6's general reference to becoming pregnant without infecting her partner.

Under the third sub-theme, participants described teaching they experienced as routine but not matched to their own situations. P2, a divorced woman who wanted more children but had no current partner, paired an approximate figure of about 70 percent with the observation that her wish for more children was constrained by the absence of a partner, a circumstance the teaching did not address. P9, a married woman with three children who regarded her family as complete, explained that although teaching was given, she did not pay attention to it because she did not want more children. Her account is notable not as an absence of engagement but as an explanation of why generalized teaching did not connect with her position.

A divergent account came from P1, a recently diagnosed single woman early in treatment. Asked whether she had received counseling on fertility and HIV, she said she had not yet, and expected that she would in due course, describing her contact with providers mainly in terms of medication. Her account differs from the pattern in the rest of the dataset, in which participants reported receiving prevention focused teaching repeatedly, and suggests that reported counseling experience varied with time since enrolment.

### 3.4 Theme 2: participants described providers as warm and accessible, while experiencing the counseling itself as not individualized

This theme concerns how participants described their interactions with staff. Illustrative accounts, prompts, and contexts are presented in Table 4.

**Table 4:** Illustrative participant accounts on interactions with providers.

Sub-theme	Interview prompt	Participant's situation	Illustrative account	Participant
Providers described as approachable and encouraging	How would you describe your experience with health care services?	Married woman describing her overall relationship with clinic staff	“I have a very cordial experience with the health workers… they granted me unlimited access to have time with them and also have time for me.”	P6 (Female, Married)
Providers described as approachable and encouraging	How would you describe your experience with health care services?	Single woman with three children, summarizing satisfaction with the service	“I am satisfied with their services. That I can still give birth any time and have a healthy baby.”	P10 (Female, Single)
Counseling experienced as pitched to assumed rather than actual intentions	How do providers address your fertility concerns?	Married man with six children who does not want more	“They give me the needed care. Yes, but I don't pay much attention because I have enough kids.”	P7 (Male, Married)
Counseling experienced as pitched to assumed rather than actual intentions	How would you describe your experience with health care services?	Single woman describing her service experience in general terms	“I have access and by taking the medications given I feel healthy.”	P8 (Female, Single)
Fertility raised routinely, but without tailoring	Have you received any counseling on fertility and HIV?	Single woman asked whether counseling was offered or had to be requested	“Yes please, every time I come for checkup.”	P8 (Female, Single)
Fertility raised routinely, but without tailoring	Have you received any counseling on fertility and HIV?	Married woman describing the regularity of counseling contact	“Yes please, the doctors and nurses have been very supportive. I am counseled each time I visit the hospital.”	P3 (Female, Married)

Asked to describe their experience of health services, participants who spoke to this theme portrayed staff in positive terms. P6 described a cordial relationship with health workers and emphasized that she was given unlimited access and time. P10 stated simply that she was satisfied with the services, linking this to confidence that she could still give birth and have a healthy baby. No participant in this dataset described being refused a discussion, spoken to dismissively, or judged for wanting children.

Participants also indicated that fertility was raised by staff as a matter of routine rather than only on request. P8 and P3 both said, when asked whether they had received counseling on fertility and HIV, that this happened at every check-up or every visit, with P3 describing doctors and nurses as very supportive.

Alongside these positive relational accounts, some participants described counseling that did not connect with their actual position. P7, a married man with six children, said that he received the needed care but did not pay much attention because he had enough children. P8's account of her service experience was brief and framed around access and medication rather than around reproductive planning. Read together, these accounts describe an experience in which staff were approachable and raised fertility regularly, while the content participants received was not experienced as matched to their individual circumstances. It should be emphasized that these are participants’ experiences of the counseling they received, and they do not establish how counseling was actually delivered or what was covered in consultations not reported here.

Because men's experiences may differ from women's, the male accounts were examined specifically. Of the three male participants, only P7 spoke at any length to fertility counseling, and he framed the topic around a completed family and economic considerations rather than future childbearing. His position aligned with that of women who considered their families complete, notably P9, suggesting that in this dataset parenting stage rather than sex was the more visible axis of difference. P4 and P11 said little in response to the fertility counseling questions, and no claim is made here about men's experiences in general on the basis of a single detailed account.

### 3.5 Theme 3: participants reported reliable treatment and routine education, but few services beyond basic counseling

This theme describes how participants experienced the organization of reproductive health within HIV care. Illustrative accounts, prompts, and contexts are presented in Table 5.

**Table 5:** Illustrative participant accounts on service organization.

Sub-theme	Interview prompt	Participant's situation	Illustrative account	Participant
Reliable ART access and reported health stability	How would you describe your experience with health care services?	Single woman linking treatment to her sense of health and future possibility	“I have access and by taking the medications given I feel healthy. That having the disease doesn't deter one from not having kids… we can still have healthy babies.”	P8 (Female, Single)
Reliable ART access and reported health stability	How do providers address your fertility concerns?	Married woman considering whether treatment would affect a future baby	“That even if I want to have a child again, taking my medications will not affect the baby.”	P9 (Female, Married)
Reproductive messaging reported as part of routine visits	Have you received any counseling on fertility and HIV?	Married woman describing the regularity of counseling within routine care	“Yes please, the doctors and nurses have been very supportive. I am counseled each time I visit the hospital.”	P6 (Female, Married)
Reproductive messaging reported as part of routine visits	What would motivate you to have a child in your condition?	Married woman describing teaching received as part of general clinic education	“We are taught at the hospital after child bearing in our condition and our general wellbeing.”	P3 (Female, Married)
Few reported services or referrals beyond education	What are your reasons for wanting or not wanting more children?	Divorced woman whose barrier to childbearing is the absence of a partner	“I will rate them about 70%. I want to have more babies but I don't have a partner at the moment.”	P2 (Female, Divorced)
Few reported services or referrals beyond education	How would you describe your experience with health care services?	Single woman summarizing what the service provided her	“I am satisfied with their services… we are taught at the hospital after child bearing in our condition.”	P10 (Female, Single)

Participants consistently described reliable access to treatment and connected it to their sense of health and to the possibility of childbearing. P8 linked having access and taking her medication to feeling healthy, and in the same account to the belief that having HIV did not prevent her from having children. P9, asked how providers addressed her fertility concerns, reported being told that taking her medication would not affect a future baby.

Reproductive messaging was described as embedded in routine visits rather than as a separate service. P6 reported being counseled each time she visited the hospital, and P3 described teaching at the hospital about childbearing in their condition and about general wellbeing, situating reproductive content within broader health education.

Accounts of anything beyond education and basic counseling were sparse. When participants described what the service offered them, they referred to teaching and general wellbeing, as in the accounts of P2 and P10, and not to fertility assessment, assisted reproduction, partner services, or onward referral. P2's account is the clearest instance: her stated barrier to childbearing was the absence of a partner, and her account contains no reference to any service or discussion addressing that circumstance. No participant in this dataset reported being referred elsewhere for a reproductive concern. This is an absence in what participants reported, and it does not establish whether such services or referral pathways existed at the facility.

## 4 Discussion

This study explored how the reproductive counseling that PLHIV reported receiving shaped their fertility decision making at a Ghanaian ART clinic, and which aspects of counseling support they perceived to be missing. Before interpreting the findings it is important to state precisely what the data are and are not. The dataset consists of what twelve patients recalled being told, how they understood it, and how they experienced their contacts with staff. It does not measure counseling quality, it does not assess what providers know or how they were trained, and it does not document what was actually said in any consultation. The interpretations that follow are therefore framed as inferences requiring further investigation rather than as demonstrated facts about services or providers.

What the data show is a consistent asymmetry in what participants reported. Education about preventing transmission to the baby was recalled repeatedly, described in concrete terms, and linked by participants themselves to greater confidence about childbearing. Guidance about conceiving safely with a partner of a different HIV status was almost entirely absent from participants’ accounts, appearing in only one interview and there only in general terms. Counseling was also experienced as pitched at people in general rather than at the participant's own relationship and parenting circumstances. These are reported experiences, and they are consistent with, though they do not by themselves demonstrate, incomplete reproductive counseling of the kind documented in other settings in the region (12–15).

The recalled strength of prevention focused education is consistent with evidence that provider delivered education supports safe childbearing intentions among PLHIV (9, 10). Interpreted through the Theory of Planned Behavior, participants who internalized that they could have healthy children were describing an increase in perceived behavioral control, the belief that one can safely carry out the behavior in question, which the theory identifies as a determinant of intention (8). This is an interpretation applied after analysis rather than a finding generated by it. That some participants remained hesitant despite accurate information is consistent with the recognized distinction between cognitive understanding and affective comfort (31), and suggests that information alone may not resolve reproductive anxieties, though the present data cannot test this.

Participants’ accounts of limited guidance on conceiving safely with a partner align with patterns documented elsewhere in the region, where the evidence comes from providers directly rather than from patients. In two Malawian HIV clinics, providers held supportive attitudes toward childbearing but lacked accurate safer conception knowledge (12), and an assessment of 57 Ugandan providers found limited knowledge, self-efficacy, and intention to provide safer conception counseling despite client childbearing desire of 59 percent and serodifferent partnerships near 50 percent (13). Patient level data show a corresponding pattern, with only 13.2 percent of 5,198 women living with HIV in Uganda able to identify three or four safer conception methods, 42 percent able to name only one, and fewer than one in six aware of sperm washing or pre exposure prophylaxis in a separate sample (14, 15). Qualitative work has described providers who felt unable to counsel confidently because they lacked tools and training (32, 33). The convergence between the present accounts and this literature is suggestive, but the direction of explanation cannot be established from patient reports, and alternative explanations are considered below.

A finding that merits particular attention is the coexistence of warmth and generality. Participants described staff as approachable, generous with time, and willing to raise fertility without being asked, and no participant reported judgment or refusal. Yet the same participants experienced the content as not matched to their situation. Supportive attitudes are known to encourage engagement, while judgmental attitudes create barriers (10, 34), and on that measure the interpersonal dimension of this service was experienced positively. The present accounts indicate that a positive interpersonal experience did not, for these participants, coincide with individualized content. This distinction is analytically important because it suggests that interventions targeting attitudes alone would not address what participants described as missing, though establishing why content was experienced as general would require investigation beyond patient reports.

Situating the findings within Ghana strengthens their interpretation. National policy positions comprehensive reproductive health support for PLHIV within HIV care (4), and studies from the Ashanti Region have documented substantial unmet need for family planning and gaps in reproductive counseling among women living with HIV at facilities in Kumasi, together with a preference among these women for discussing reproductive concerns with their HIV clinicians (20–22). The present accounts extend that largely quantitative, contraception focused regional evidence by showing, in patients’ own words, that the gap they perceive lies less in prevention of transmission to the infant and more in safer conception and individualized planning. Convergence across facilities in the same region suggests the pattern may not be confined to one clinic, while differences in facility type, ownership, and method caution against generalization.

The reported reliability of treatment access, and the embedding of reproductive messaging within routine visits, are consistent with recommended practice in comprehensive HIV care (9, 35). Reliable access appeared in participants’ accounts as the foundation on which positive fertility intentions rested, which accords with evidence that effective treatment reshapes life possibilities including parenthood (6, 7). Evidence from implementation elsewhere indicates that where structured safer conception services are established, PLHIV take them up and continue them, with continuation of self-insemination at 69.1 percent and timed condomless sex at 75.4 percent of follow up visits at a South African demonstration clinic (16). This suggests that patient willingness is unlikely to be the binding constraint on the components participants described as absent.

Several explanations for the pattern participants described should be weighed, and they extend well beyond individual provider knowledge. International guidance sets out the range of options for achieving pregnancy safely in serodifferent relationships, including viral suppression through ART, oral pre exposure prophylaxis for the partner without HIV, timing intercourse away from peak fertility, screening and treatment of sexually transmitted infections, and manual insemination, and it recommends that sexual and reproductive health services be integrated within HIV services (36). Delivering this range within a routine consultation requires time, protocols, commodities, and defined referral routes, any of which may be constrained irrespective of what an individual member of staff knows. Short consultations, high patient volumes, the absence of a standardized counseling protocol, and the availability of onward referral are all plausible contributors to counseling being experienced as general.

The institutional setting is a further consideration that requires explicit attention. St. Michael's Hospital is a Catholic mission facility operating within the Catholic Health Service of Ghana. Evidence from faith based health systems elsewhere in sub Saharan Africa indicates that institutional norms shape which reproductive topics can be addressed. A qualitative study of doctors at three Catholic hospitals in Cameroon found that clinicians navigated explicit restrictions on discussing and providing modern contraception, described referral as their principal workaround, and reported that these constraints narrowed the sexual and reproductive health care they could offer (37). Quantitative analysis of family planning consultations in Malawi similarly found systematic differences between faith based and public providers in the content of reproductive health service delivery (38). Faith based providers deliver a substantial share of health care across the region and are central to HIV service provision, so this is not a marginal consideration. In the present study, participants did not raise religious restrictions, and no inference is drawn about the policies of the study facility. The point is rather that institutional context is a plausible influence on the scope of reproductive counseling in such settings, and it must be weighed alongside provider level explanations. Determining whether and how it operated here would require direct study of the facility's policies and of the staff delivering counseling.

Recommendations arising from this study are confined to the specific areas participants themselves identified. First, participants recalled detailed education on preventing transmission to the infant but almost no guidance on conceiving safely with a partner of a different HIV status; the indicated area for strengthening is therefore safer conception counseling specifically, covering the options set out in international guidance, rather than general awareness training (13, 32, 33, 36). Second, participants experienced counseling as pitched at people in general rather than at their circumstances, and two participants described disengaging from it for that reason; a brief structured prompt that establishes each patient's fertility intentions and relationship situation before counseling is given would address this specific experience (32). Third, participants reported reproductive messaging as already embedded in routine visits, so the indicated direction is to extend the content of an existing integrated contact rather than to create a separate service (9, 17, 35, 36). Fourth, no participant reported any referral for a reproductive concern, and one participant's stated barrier, the absence of a partner, went unaddressed in her account; clearer and documented referral pathways for concerns falling outside routine counseling are therefore indicated. Fifth, the gap between participants describing staff as warm and simultaneously experiencing counseling as generic points to communication practice, specifically eliciting and responding to the individual's stated intentions. These are areas for strengthening identified from participant accounts; whether current practice differs from what participants recalled, and what would be required to change it, remain open questions.

### 4.1 Strengths and limitations

The study's principal strength is that it documents, in patients’ own words, how PLHIV experience reproductive counseling and what they perceive to be missing, complementing a regional evidence base that is largely quantitative and provider focused. The design, analysis, and reporting are internally consistent, the analysis was inductive with independent second coding, and positionality was examined systematically (24–26, 29, 30).

Several limitations bound what can be concluded. First, and most importantly, healthcare providers were not included. No provider was interviewed, observed, or assessed, and no clinical records or counseling protocols were examined. The study therefore cannot speak to provider knowledge, training, or preparedness, and any statement about these would exceed the evidence. Confirming whether the gaps participants perceived correspond to gaps in what is delivered would require research that engages providers directly.

Second, the findings reflect reported experience rather than objective measurement. What participants recalled being told is shaped by memory, comprehension, salience, and the passage of time. Information may have been provided and forgotten, provided and misunderstood, or understood differently from how it was intended. The absence of safer conception content from participants’ accounts is therefore an absence in what was recalled and reported, which is not equivalent to an absence in what was delivered.

Third, the study was conducted at a single site. One district level clinic, with approximately 120 registered PLHIV, cannot represent the range of ART service delivery in Ghana. The facility is also a Catholic mission hospital, and evidence from comparable faith based settings indicates that institutional norms can shape the scope of reproductive health services (37, 38). Findings may therefore differ from those that would emerge in public sector, secular non governmental, or tertiary facilities, and transferability is limited to comparable district level, faith based, or resource constrained ART settings.

Fourth, selection bias is likely. Participants were patients who were willing to discuss fertility, sexuality, and their experience of care with a researcher. Those who declined most often cited reluctance to discuss fertility, so the sample probably over represents people comfortable with the topic and engaged with the clinic, and under represents those who are disengaged, more private, or more dissatisfied. The exact number approached and declining was not recorded, which prevents assessment of the extent of this bias.

Fifth, interviews took place at the clinic on which participants depended for treatment, and were conducted by a health professional. Despite the safeguards described in Section 2.7, this asymmetry may have encouraged socially desirable responses and discouraged criticism of staff. The uniformly positive accounts of interpersonal treatment should be read with this in mind, and dissatisfaction may be understated.

Sixth, contribution to the themes was uneven. Four participants, including two of the three men and the widowed participant, spoke little to the counseling questions, so the findings rest disproportionately on a subset of the sample. Men's experiences in particular are represented by a single detailed account and should not be generalized.

Seventh, some reporting is incomplete. Time since diagnosis was not systematically documented and is not reported, which prevents examination of whether counseling experience varied with duration in care, a question the divergent account of the recently diagnosed participant makes pertinent. Transcripts were not returned to participants for comment, and no participant validation of themes was undertaken.

### 4.2 Conclusion

Participants at a Ghanaian ART clinic reported clear and repeated education about preventing HIV transmission to a baby, and described staff as warm, accessible, and willing to raise fertility without being asked. They reported almost no guidance on conceiving safely with a partner of a different HIV status, experienced counseling as pitched at people in general rather than at their own circumstances, and described no services or referrals beyond education and basic counseling. These are patients’ reported experiences and perceptions. They do not measure counseling quality or provider knowledge, and the pattern may equally reflect consultation time, service organization, referral availability, and the faith based institutional context, all of which require direct investigation. What the accounts do identify, from the patient standpoint, are specific areas where participants perceived their needs to be unmet: safer conception counseling for serodifferent relationships, fertility planning matched to individual circumstances, and clearer routes to support for concerns falling outside routine teaching. Establishing whether and how these areas can be strengthened will require research that includes providers and examines service organization alongside patient experience.

## Data Availability

The qualitative datasets generated and analysed during this study contain potentially identifying participant information and are therefore not publicly available, in order to protect participant confidentiality. De-identified data may be made available by the corresponding author on reasonable request and subject to ethical approval.

## Supplementary material

Supplementary Material S1 Interview guide.
