# Supplementary material for: Fertility counseling experiences and perceived gaps in provider support among people living with HIV: a qualitative study from an antiretroviral therapy clinic in Ghana
Source: Front Reprod Health. 2026 Aug 18;8:1914832. doi: 10.3389/frph.2026.1914832 (PMC13529651; doi:10.3389/frph.2026.1914832)
Supplement: Supplementary file 1 [file Supplementaryfile1.docx]

**Supplementary Material**

**De-Identified Qualitative Excerpts and Coded Thematic Summaries**

**Manuscript:** Inadequate Provider Training and Knowledge Gaps in Fertility Counseling for People Living with HIV: A Qualitative Study from Ghana

**S1. Note on De-Identification**

All excerpts were extracted from verbatim interview transcripts and de-identified before analysis and reporting. Participant names were replaced with sequential codes (P1 to P12). Only non-identifying attributes (sex, marital status, and HIV-positive status) are retained to support interpretation. Place names, facility-specific references, partner names, and any incidental personal details that could enable identification were removed or generalized. Audio files and master code lists were stored separately on password-protected devices accessible only to the research team. Excerpts are presented as spoken, with minor elisions marked by ellipses where unrelated content was removed for confidentiality or concision.

**S2. Coding Framework**

Codes progressed from descriptive, in-vivo initial codes that stayed close to participants' own language, through focused analytic codes, to the three main themes reported in the manuscript. The framework below maps each theme and sub-theme to its focused codes.

| **Theme** | **Sub-theme** | **Focused codes** |
| --- | --- | --- |
| 1. Quality of Reproductive Health Counseling and Education | 1.1 Comprehensive PMTCT Education | PMTCT knowledge gained; healthy-baby reassurance; ART safety in pregnancy; routine education at visits |
|  | 1.2 Safer Conception Counseling for Serodiscordant Couples | partner protection knowledge; conception without infecting partner; gap in serodifferent counseling; PMTCT-only framing |
|  | 1.3 Individualized Reproductive Health Planning | tailored vs general counseling; below-excellence rating; unmet circumstance-specific need; disengagement from generic message |
| 2. Healthcare Provider Attitudes and Support | 2.1 Supportive and Encouraging Provider Approaches | provider accessibility; time and rapport; non-judgmental support; satisfaction with services |
|  | 2.2 Perceived Judgmental or Generalized Attitudes | assumption based on parity; assumption based on marital status; selective attention; generalized counseling |
|  | 2.3 Provider Comfort with Fertility Discussions | proactive provider initiation; counseling at every visit; routine engagement; variation in initiative |
| 3. Access to Comprehensive Reproductive Health Services | 3.1 ART Access and Adherence Support | reliable ART access; health stability; ART compatible with healthy pregnancy |
|  | 3.2 Integration of Reproductive Health into HIV Care | counseling embedded in routine care; reproductive health within wellness education; systematic delivery |
|  | 3.3 Availability of Specialized Reproductive Services | education without specialized service; limited referral pathways; unmet specialized need |

**S3. De-Identified Excerpts and Coded Thematic Summaries**

**Theme 1. Quality of Reproductive Health Counseling and Education**

**Sub-theme 1.1 Comprehensive PMTCT Education**

| **Excerpt** | **Participant** | **Codes** |
| --- | --- | --- |
| "That having the disease doesn't deter one from not having kids and in our condition, we can still have healthy babies... every time I come for checkup. I am just hopeful that when the time comes for me to have a baby I can also go through my due months and have a healthy baby as any other woman out there." | P8 (Female, HIV+, Single) | healthy-baby reassurance; routine education at visits |
| "I was told that you will be put on medications to protect the unborn baby from getting infected. We are taught at the hospital after child bearing in our condition and our general wellbeing. It has changed my perspective on marriage and child bearing." | P3 (Female, HIV+, Married) | PMTCT knowledge gained; ART safety in pregnancy |
| "That even if I want to have a child again, taking my medications will not affect the baby. Yes please, we are taught but I don't need babies again so I don't really pay attention to it." | P9 (Female, HIV+, Married) | ART safety in pregnancy; disengagement from generic message |

*Coded thematic summary:* PMTCT education emerged as a consistent strength of the service. Participants reported repeated, concrete information that ART can protect an unborn child, and several explicitly stated that this knowledge reshaped their fertility intentions in a positive direction. The recurrence of healthy-baby reassurance and ART safety codes indicates that providers were well prepared to deliver core vertical transmission messaging. The disengagement code in P9's account signals that even strong messaging was not always tailored to individual intentions.

**Sub-theme 1.2 Safer Conception Counseling for Serodiscordant Couples**

| **Excerpt** | **Participant** | **Codes** |
| --- | --- | --- |
| "There are drugs I can take to prevent my baby from having the disease and how I can get pregnant with my partner without infecting him as well... I am counselled each time I visit the hospital." | P6 (Female, HIV+, Married) | partner protection knowledge; conception without infecting partner |
| "That I can still give birth any time and have a healthy baby. Yes please, we are taught at the hospital after child bearing in our condition and our general wellbeing." | P10 (Female, HIV+, Single) | PMTCT-only framing; gap in serodifferent counseling |

*Coded thematic summary:* Counseling that extended beyond vertical transmission to partner protection was the exception rather than the rule. P6's account, coded for conception without infecting partner, demonstrates that comprehensive safer conception content was occasionally delivered. However, most descriptions, such as P10's, carried the PMTCT-only framing code and centered on childbearing and general wellbeing without addressing horizontal transmission. The predominance of the gap code in this sub-theme is consistent with limited provider preparation in safer conception methods.

**Sub-theme 1.3 Individualized Reproductive Health Planning**

| **Excerpt** | **Participant** | **Codes** |
| --- | --- | --- |
| "We are being taught every time I visit the hospital and I will rate them about 70%. I want to have more babies but I don't have a partner at the moment." | P2 (Female, HIV+, Divorced) | below-excellence rating; unmet circumstance-specific need |
| "Yes please, we are taught but I don't need babies again so I don't really pay attention to it." | P9 (Female, HIV+, Married) | tailored vs general counseling; disengagement from generic message |

*Coded thematic summary:* Counseling was routine but not individualized. The below-excellence rating and unmet circumstance-specific need codes in P2's account indicate that standardized messaging did not address her situation as a divorced woman without a current partner. The disengagement code in P9's account reflects counseling that did not adapt to a completed family. Together, these codes point to a standardized approach that fell short of individualized reproductive planning.

**Theme 2. Healthcare Provider Attitudes and Support**

**Sub-theme 2.1 Supportive and Encouraging Provider Approaches**

| **Excerpt** | **Participant** | **Codes** |
| --- | --- | --- |
| "I have a very cordial experience with the health workers, I am saying so because they granted me unlimited access to have time with them and also have time for me... I am counselled each time I visit the hospital." | P6 (Female, HIV+, Married) | provider accessibility; time and rapport |
| "I am satisfied with their services. That I can still give birth any time and have a healthy baby." | P10 (Female, HIV+, Single) | satisfaction with services; non-judgmental support |

*Coded thematic summary:* The relational quality of provider interactions was largely positive. Provider accessibility, time and rapport, and satisfaction codes recurred across accounts, indicating an environment in which fertility could be discussed. Supportive attitudes were a clear asset, though they did not, on their own, guarantee depth of content.

**Sub-theme 2.2 Perceived Judgmental or Generalized Attitudes**

| **Excerpt** | **Participant** | **Codes** |
| --- | --- | --- |
| "They give me the needed care. Yes, but I don't pay much attention because I have enough kids." | P7 (Male, HIV+, Married) | assumption based on parity; selective attention |
| "I have access and by taking the medications given I feel healthy. Health is most important to me when considering whether to have children." | P8 (Female, HIV+, Single) | assumption based on marital status; generalized counseling |

*Coded thematic summary:* This sub-theme surfaced a subtler limitation. The assumption-based codes suggest that counseling intensity may have tracked parity or marital status rather than individual intentions. While no account described overt judgment, the selective attention and generalized counseling codes indicate counseling that was not consistently individualized, showing that supportive attitudes coexisted with shallow content.

**Sub-theme 2.3 Provider Comfort with Fertility Discussions**

| **Excerpt** | **Participant** | **Codes** |
| --- | --- | --- |
| "Yes please, every time I come for checkup. That having the disease doesn't deter one from not having kids and in our condition, we can still have healthy babies." | P8 (Female, HIV+, Single) | proactive provider initiation; counseling at every visit |
| "Yes please, the doctors and nurses have been very supportive. I am counselled each time I visit the hospital. We are taught at the hospital after child bearing in our condition and our general wellbeing." | P3 (Female, HIV+, Married) | routine engagement; counseling at every visit |
| "They give me the needed care. Yes, but I don't pay much attention because I have enough kids." | P7 (Male, HIV+, Married) | variation in initiative; selective attention |

*Coded thematic summary:* Provider comfort in raising fertility topics appeared adequate. Proactive initiation and counseling-at-every-visit codes recurred, indicating that fertility was addressed without waiting for patients to raise it. The variation in initiative code in P7's account shows that routine offering did not always align with patient receptiveness, again pointing to a need for tailoring rather than a lack of comfort.

**Theme 3. Access to Comprehensive Reproductive Health Services**

**Sub-theme 3.1 ART Access and Adherence Support**

| **Excerpt** | **Participant** | **Codes** |
| --- | --- | --- |
| "I have access and by taking the medications given I feel healthy. That having the disease doesn't deter one from not having kids... we can still have healthy babies." | P8 (Female, HIV+, Single) | reliable ART access; health stability |
| "They give me the needed care. That even if I want to have a child again, taking my medications will not affect the baby." | P9 (Female, HIV+, Married) | ART compatible with healthy pregnancy |

*Coded thematic summary:* ART access functioned as the foundation for positive fertility intentions. Reliable access and health stability codes recurred, and the ART-compatible-with-pregnancy code indicates that participants understood treatment as supporting rather than precluding childbearing. This foundational element of the service was robust.

**Sub-theme 3.2 Integration of Reproductive Health into HIV Care**

| **Excerpt** | **Participant** | **Codes** |
| --- | --- | --- |
| "Yes please, every time I come for checkup... I am counselled each time I visit the hospital." | P6 (Female, HIV+, Married) | counseling embedded in routine care; systematic delivery |
| "We are taught at the hospital after child bearing in our condition and our general wellbeing. It has changed my perspective on marriage and child bearing." | P3 (Female, HIV+, Married) | reproductive health within wellness education |

*Coded thematic summary:* Basic reproductive messaging was embedded within routine HIV care. The embedded-in-routine-care and systematic-delivery codes indicate that counseling was offered at every visit as part of general wellness education. Integration of basic education was achieved, providing a platform on which more individualized provision could be built.

**Sub-theme 3.3 Availability of Specialized Reproductive Services**

| **Excerpt** | **Participant** | **Codes** |
| --- | --- | --- |
| "We are being taught every time I visit the hospital and I will rate them about 70%. I want to have more babies but I don't have a partner at the moment. I am hesitant in child bearing but hopefully one day I will follow all the processes so I can get a healthy baby." | P2 (Female, HIV+, Divorced) | education without specialized service; unmet specialized need |
| "I am satisfied with their services... we are taught at the hospital after child bearing in our condition and our general wellbeing." | P10 (Female, HIV+, Single) | education without specialized service; limited referral pathways |

*Coded thematic summary:* The accounts in this sub-theme focused on education rather than specialized clinical services. The education-without-specialized-service code recurred, and the unmet specialized need and limited referral pathways codes indicate that the service did not extend to fertility evaluation, assisted reproduction, or structured support for participants in particular circumstances. This marks the upper limit of provision and is consistent with constrained specialized training and capacity.

**S4. Summary of Coded Patterns Across Themes**

| **Dimension** | **Strength of provision** | **Coded evidence of gap** |
| --- | --- | --- |
| Prevention of mother to child transmission | Strong and routine | Disengagement when not tailored (P9) |
| Safer conception for serodifferent couples | Weak and inconsistent | PMTCT-only framing; gap in serodifferent counseling (P10) |
| Individualized planning | Limited | Below-excellence rating; unmet circumstance-specific need (P2) |
| Provider attitude and comfort | Strong | Assumption-based, generalized counseling (P7, P8) |
| ART access and basic integration | Strong | None of substance |
| Specialized services and referral | Weak | Education without specialized service; limited referral (P2, P10) |

Across themes, the coded evidence shows an asymmetry in which prevention messaging, provider rapport, treatment access, and basic integration were strong, while safer conception counseling, individualized planning, and specialized provision were weak. The gap codes cluster precisely in the domains that depend most on specialized provider knowledge and training, supporting the manuscript's central interpretation.
